# From Blood to Lesioned Brain: An In Vitro Study on Migration Mechanisms of Human Nasal Olfactory Stem Cells

**DOI:** 10.1155/2017/1478606

**Published:** 2017-06-18

**Authors:** Stéphane D. Girard, Isabelle Virard, Emmanuelle Lacassagne, Jean-Michel Paumier, Hanae Lahlou, Françoise Jabes, Yves Molino, Delphine Stephan, Kevin Baranger, Maya Belghazi, Arnaud Deveze, Michel Khrestchatisky, Emmanuel Nivet, François S. Roman, François Féron

**Affiliations:** ^1^Aix Marseille Univ, CNRS, NICN, Marseille, France; ^2^VECT-HORUS SAS, Faculty of Medicine, Marseille, France; ^3^Aix Marseille Univ, CNRS, CRN2M, Marseille, France; ^4^Aix Marseille Univ, IFSTTAR, LBA, Marseille, France; ^5^APHM, CHU Nord, ENT Department, Marseille, France; ^6^APHM, Culture and Cell Therapy Laboratory, CIC BT 1409, Marseille, France

## Abstract

Stem cell-based therapies critically rely on selective cell migration toward pathological or injured areas. We previously demonstrated that human olfactory ectomesenchymal stem cells (OE-MSCs), derived from an adult olfactory lamina propria, migrate specifically toward an injured mouse hippocampus after transplantation in the cerebrospinal fluid and promote functional recoveries. However, the mechanisms controlling their recruitment and homing remain elusive. Using an in vitro model of blood-brain barrier (BBB) and secretome analysis, we observed that OE-MSCs produce numerous proteins allowing them to cross the endothelial wall. Then, pan-genomic DNA microarrays identified signaling molecules that lesioned mouse hippocampus overexpressed. Among the most upregulated cytokines, both recombinant SPP1/osteopontin and CCL2/MCP-1 stimulate OE-MSC migration whereas only CCL2 exerts a chemotactic effect. Additionally, OE-MSCs express SPP1 receptors but not the CCL2 cognate receptor, suggesting a CCR2-independent pathway through other CCR receptors. These results confirm that OE-MSCs can be attracted by chemotactic cytokines overexpressed in inflamed areas and demonstrate that CCL2 is an important factor that could promote OE-MSC engraftment, suggesting improvement for future clinical trials.

## 1. Introduction

In both acute injury and neurodegenerative disorders of the adult central nervous system (CNS), intrinsic regenerative capacities usually fail to compensate neuronal loss. Therefore, exogenous cell therapy is developed as a novel treatment, where transplanted cells may replace dead cells, act as neurotrophic or neuroprotective agents, or deliver biotherapeutic molecules [1]. Transplanted cells derived either from embryonic stem cells, induced pluripotent stem cells, or neural stem/progenitor cells have shown great promises in various models of cerebral pathology [2–4]. However, problems often arose, including ethical issues, cell availability, graft rejection, and risk of tumor formation [5–7]. Thus, testing alternative cell types remains of great interest, especially adult peripheral stem cells [8].

Adult stem cells from the human nasal olfactory mucosa, a peripheral and permanently self-renewing nervous tissue, stand as promising candidates [9–11]. We characterized them as multipotent mesenchymal stem cells with neurogenic properties and named them olfactory ectomesenchymal stem cells (OE-MSCs) [11]. Beyond their capacity to generate neural cells, other properties support their potential usefulness for autologous stem cell-based therapies: easily accessible in every individual [12], they proliferate at a high rate in vitro, while they do not seem to form tumors after transplantation [11, 13]. In rodents, OE-MSCs successfully improved models of myocardial infarct [14], spinal cord trauma [15–17], cochlear damage [18, 19], or Parkinson's disease [20]. We demonstrated their therapeutic potential in a mouse model of excitotoxically induced neuronal death that mimics an ischemic/hypoxic injury in the hippocampus [13]. We showed that human OE-MSCs survive after intracerebral transplantation and promote learning and memory recovery. Interestingly, they migrate specifically toward the lesioned hippocampus after transplantation into either the controlateral unlesioned side or the cerebrospinal fluid (CSF) [13]. Moreover, this directed migration and cognitive recovery can take place four weeks after the lesion, a delay required for expanding high numbers of OE-MSCs from an individual in the prospect of an autologous graft [11].

Though it is very efficient to graft a large number of cells into the desired brain area, transplantations into the brain tissue or the CSF represent risky interventions, especially in aged or fragile individuals. Systemic transplantation, into either veins or arteries, constitutes a less invasive approach (for reviews: [21, 22]). An increasing number of studies, including clinical trials, report intravenous or intra-arterial transplantation of mesenchymal stem cells against CNS lesions or disorders [23]. Thus, selective migration toward a pathological or traumatized area is a critical step in stem cell regenerative medicine. For effective therapy, stem cell homing is necessary to reduce migration to other areas while allowing the delivery of stem cells via less invasive routes and, possibly, excluding unwanted side effects [24]. Many studies demonstrated the tropism of both endogenous and transplanted stem/progenitor cell types for inflamed tissues, including hypoxic-ischemic areas, glial tumors, and other injury-associated zones where neuroinflammatory responses involve components of the innate immune system [25–29]. Inflammation strongly upregulates chemotactic cytokines in cerebral pathologic areas, and these molecules have been implicated in the migration of immune and stem cells to these sites [24]. Identifying the molecular pathways directing stem cell migration might be crucial for improving therapeutic intervention in several neurological diseases [30]. We recently demonstrated that OE-MSCs strongly express matrix metalloproteinases (MMPs) such as MMP2, MMP9, and MT1-MMP and showed their importance in OE-MSC migration [31]. However, the molecular mechanisms regulating OE-MSC attraction and homing to injured brain areas have not yet been investigated.

The present study focuses on how olfactory cells transplanted into the circulation manage to reach the lesioned brain, crossing first the blood-brain barrier (BBB) and then migrating through the nervous parenchyma. To better understand the cellular and molecular mechanisms at play, we used an experimental approach based on in vitro models of BBB, an exhaustive proteomic analysis of proteins secreted by OE-MSCs, a pan-genomic microarray study of molecules released by lesioned cells and cell migration assays.

## 2. Materials and Methods

### 2.1. Cell Culture

Following a protocol approved by the local ethical committee (Comité de Protection des Personnes, Marseille, France, file number 205 016), human nasal olfactory mucosae were obtained by biopsy during routine nasal surgery under general anesthesia. Biopsies were immediately placed in growth medium containing DMEM/Ham F12 supplemented with 10% fetal bovine serum (FBS) and 100 units/mL of penicillin and 100 *μ*g/mL of streptomycin (Invitrogen/Life Technologies). OE-MSCs were purified from the lamina propria and cultured as described before [11, 13]. All experiments were performed using OE-MSCs obtained after 4 to 12 passages from the initial cultures. OE-MSCs from three different donors were used in this study. When needed, OE-MSCs were infected with a GFP lentiviral vector. The acute monocytic leukemia cell line THP1 used as positive control in RT-qPCR experiments was obtained from the American Type Culture Collection and cultured in RPMI 1640 containing 10% FBS and 100 units/mL of penicillin and 100 *μ*g/mL of streptomycin.

### 2.2. Blood-Brain Barrier (BBB) Model and Permeability Test

To analyze OE-MSC transmigration, we used a model of BBB with two compartments separated by a porous membrane (pore size: 1 *μ*m), already described by Molino et al. [32]. Briefly, rat endothelial cells from blood cerebral microvessels were cultured in the top compartment on polyethylene insert filters coated with collagen IV and fibronectin, at a cell density of 500,000 per insert in 6-well culture plates. Rat glial cells were cultured in the bottom compartment at a density of 16,000 cells/cm^2^. For both compartments, the culture medium was composed of DMEM/Ham F12 supplemented with 20% bovine serum depleted in platelets, 2 ng/mL fibroblast growth factor 2 (FGF2), and 500 nM hydrocortisone [32]. For each experiment, 3 to 4 conditions were evaluated in duplicate: 0, 120,000, and 500,000 OE-MSCs were seeded on top of the endothelial cells, then incubated for 24 h. Permeability to the Lucifer yellow dye (LY, Sigma-Aldrich L0259) was next measured over 60 min using fluorimetry, as described [32].

### 2.3. OE-MSC Secretome Analysis

We collected the conditioned medium (CM) of OE-MSCs cultured for 3 days at confluency in T75 flasks containing 10 mL of serum-free DMEM/Ham F12 with 1% insulin-transferrin-selenium-ethanolamine (ITS-X, Invitrogen/Life Technologies) and penicillin/streptomycin. One culture flask containing only serum-free medium served as negative control. The CM was filtered through sterile 0.2 *μ*m filters; 1% protease inhibitor cocktail (Calbiochem 599131) was added, and the CM was immediately frozen at −80°C. This procedure was repeated for three more days, and the CMs were combined. The CMs were concentrated and desalinated in Ultracel 3-K Amicon ultracentrifugal filters with a first centrifugation at 3220*g* during 30 min and, after adding 10 mL of sterile water (B. Braun), a second centrifugation at 3,220*g* for 20 min. Of the obtained 500 *μ*L of concentrated CM, 50 *μ*L were loaded onto a polyacrylamide gel (4–20% mini-Protean TGX gel, Bio-Rad 456-1096) and the proteins separated by electrophoresis were stained with EZ Blue staining reagent (Sigma-Aldrich G1041). After elution from the gel, peptides were obtained by trypsin hydrolysis, separated by HPLC (Ultimate 3000 Rapid Separation LC Systems, Dionex), and sequenced by tandem mass spectrometry (Q-Exactive Thermofischer). Secreted proteins were identified using the Swiss Prot data bank (http://web.expasy.org/docs/swiss-prot_guideline.html) and analyzed using the Ingenuity Pathway Analysis software (https://www.qiagenbioinformatics.com/products/ingenuity-pathway-analysis/).

### 2.4. Hippocampal Excitotoxic Lesions in Mice

Animal experiments were approved by the Ethics Committee of the Medical Faculty of Marseille and were carried out in accordance with the guidelines published in the European Communities Council Directive of November 24, 1986 (86/609/EEC). All efforts were made to minimize animal suffering and to reduce the number of mice. Unilateral or bilateral lesions of the hippocampus on ten-week-old male Balb/c mice (Charles River, L'Arbresle, France) were performed using ibotenic acid (Sigma-Aldrich, Saint-Quentin-Fallavier, France), an NMDA agonist, leading to the loss of neuronal cells in the sites of injection by cellular excitotoxicity, as described in our previous study [13]. One day after the lesion, hippocampal lesion efficiency was controlled using magnetic resonance imaging (MRI), as previously described [13].

### 2.5. Microarray Experiments

Four weeks after ibotenic acid-induced injury, bilaterally lesioned mice (*n* = 3) and control mice (*n* = 3) were decapitated. Brains were quickly removed, and hippocampi were dissected under RNAse-free conditions before being immediately placed in RNA later (Qiagen, Courtaboeuf, France) and stored at −80°C. Total RNA was isolated and treated with DNAse using the RNeasy Lipid Tissue Mini Kit (Qiagen). RNA concentration and purity were determined using a NanoDrop-1000 Spectrophotometer (NanoDrop Technologies, Thermo Fisher Scientific, Illkirch-Graffenstaden, France). RNA quality was assessed with an Agilent 2100 Bioanalyser (Agilent Technologies, Massy, France). For each sample, cDNA was generated from 600 ng of total RNA with an Agilent Quick Amp Kit (Agilent Technologies). Then complementary RNA was synthesized, amplified, and labeled with cyanine 3 dye according to the manufacturer's protocols. Cyanine 3-labeled cRNA (1.65 *μ*g) was fragmented and hybridized to the Agilent Whole Mouse Genome Oligo Microarray 4x44k at 65°C for 17 hours. After washing, fluorescence intensity at each spot was assayed using a G2565BA Microarray Scanner (Agilent). After extraction with Feature Extraction Software 9.5.3 (Agilent), data were normalized (background subtraction and quantile normalization) using AgiND library developed under R software for Agilent microarray data normalization and visualization. A fold change ratio (each lesioned hippocampus against pooled control hippocampus) was calculated for every spot. Data are available on the ArrayExpress database (accession number E-MEXP-2682).

### 2.6. Real-Time Quantitative PCR

Four weeks after injury, overexpression of cytokine candidates was assessed using real-time quantitative PCR (RT-qPCR) in control (*n* = 5) and lesioned (*n* = 6) mice. According to the manufacturer's protocol (Invitrogen/Life Technologies, Saint Aubin, France), 1 *μ*g total RNA from hippocampi (prepared as described above) was submitted to reverse transcription using hexanucleotides and Moloney murine leukemia virus (MMLV) reverse transcriptase. Quantitative PCR experiments were carried out with the 7500 Fast Real-Time PCR System (Applied Biosystems/Life Technologies). All reactions were performed using TaqMan Fast Universal PCR Master Mix and TaqMan® Gene Expression Assay probes (Applied Biosystems, see Supplementary Table 2 available online at https://doi.org/10.1155/2017/1478606 for assay IDs). For each experiment, 25 ng of previously prepared hippocampus cDNA were used. Samples were run in duplicates and analyzed with the 7500 Software v2.0 (Applied Biosystems). Relative expression levels were determined according to the ΔΔCt method, the *Gapdh* gene serving as endogenous control for normalization. Receptor expression in human OE-MSCs was assessed with the same protocol (see Supplementary Table 2 for assay IDs). In this case, data were normalized using the human housekeeping gene *ABELSON*, as previously described [33]. For each OE-MSC donor, RNA extractions were performed from three independent cultures and the reported values are the mean of these three independent experiments, each performed in duplicate.

### 2.7. Tissue Processing

One month after surgery, unilaterally injured mice (*n* = 3) were deeply anesthetized (sodium pentobarbital, i.p.) and transcardially perfused (saline then 4% paraformaldehyde). Brains were extracted, postfixed overnight in cold paraformaldehyde, and transferred to a 30% sucrose solution before being processed for cryostat sectioning (Leica Microsystems, Wetzlar, Germany). Coronal sections (35 *μ*m thick) were serially collected and kept floating at −20°C in cryoprotectant (30% glycerol, 30% ethylene glycol in 0.05 M phosphate-buffered saline (PBS)) until processed for immunostaining.

### 2.8. Immunostaining

For tissue staining, after washing in PBS, floating sections were incubated 1 hour at room temperature (RT) with blocking buffer (3% bovine serum albumin, 0.1% Triton X-100 in PBS) and overnight at 4°C with the following primary antibodies diluted in blocking solution: mouse monoclonal anti-GFAP (1/300, Millipore/Chemicon, Molsheim, France) or rabbit anti-Iba1 (1/200, Wako Pure, Chemical Industries, Osaka, Japan). Then, slices were rinsed (3 × 5 min) in PBS and incubated for 90 min at RT with cross-adsorbed AlexaFluor 488- or 594-conjugated anti-rabbit or anti-mouse secondary antibodies (1/500, Jackson Immunoresearch, West Grove, PA, USA) in the dark. After several washes in PBS, slices were counterstained with 0.5 *μ*g/mL Hoechst blue (#33342, Sigma-Aldrich) for 30 min at RT and mounted with ProLong Gold Antifade reagent (Invitrogen/Life Technologies). Images were acquired with an inverted Axio Observer microscope (Zeiss, Jena, Germany) equipped with DAPI, FITC, and rhodamine epifluorescence filters, using the mosaic mode of the Axiovision software (Zeiss).

For cytochemistry on the BBB model, OE-MSC transmigration was carried out on the 12-well plate insert filters and cells were fixed for 15 min in 4% paraformaldehyde. After three washes, filters with the cells were gently dissociated from the plastic inserts with a razor blade and incubated for 1 hour at RT with a blocking buffer and, overnight at 4°C, with the mouse anti-Claudin-5 primary antibody (1/100, Invitrogen), diluted in the blocking solution. Cells were incubated with the appropriate fluorescent secondary antibody for 1 hour at RT (1/500, Alexa Fluor 594-conjugated anti-mouse) while nuclei were labeled with Hoechst blue. Cells were washed and mounted in Prolong Gold antifade reagent.

### 2.9. Chemotaxis Assay in Modified Boyden Chambers

OE-MSCs, cultured in serum-containing medium, were detached by trypsin/EDTA, counted, and seeded into the upper chamber of cell culture inserts for 24-well plates with translucent polyethylene terephthalate (PET) filter membranes with 8 *μ*m diameter pores (BD Falcon™, BD Biosciences, Le Pont de Claix, France) at a density of 1.2 × 10^4^ cells per insert, in a final volume of 200 *μ*L of serum-free medium with 1% ITS-X. Cells were allowed to migrate through the membrane filter after cytokines were added to the lower chamber at various concentrations (human recombinant C3A and mouse recombinant SPP1, R&D systems; human recombinants CXCL10, CCL2, and SPP1, Peprotech; and mouse recombinant SPP1, Invitrogen/Life Technologies) in a final volume of 400 *μ*L of the same serum-free medium. Control inserts were prepared by substituting each cytokine with its reconstitution buffer. After 12 h of incubation (37°C in 5% CO_2_), inserts were delicately rinsed in PBS, OE-MSCs were fixed with 4% paraformaldehyde, and their nuclei were stained with 1 *μ*g/mL Hoechst blue (#33242, Invitrogen/Life Technologies). Membrane filters were observed using a Nikon E800 (Nikon, Champigny-sur-Marne, France) upright microscope equipped with epifluorescence and DAPI filter and an Orca-ER CCD camera (Hamamatsu Photonics, Hamamatsu, Japan). For quantitative assessment, the number of stained cells on each side of the membrane was manually counted and the percentage of migrating cells was calculated per field. Forty random fields were analyzed per membrane filter, using a 40x objective. Reported values are the mean percentage of migrating cells per membrane of at least three independent experiments performed in triplicate. Random cell motility (chemokinesis) was assessed by adding an equal concentration of cytokines in both upper and lower wells of the chamber.

### 2.10. Agarose Spot Assay

In order to quantify cytokine effect on OE-MSC migration, we also performed agarose spot assay, as previously described [34, 35] with few alterations. Low-melting point agarose (Ultrapure™ low-melting agarose, Invitrogen/Life Technologies) was diluted into sterile PBS in order to make a 1% agarose solution. This solution was heated until all agarose particles were dissolved and then cooled down to 37°C. Preheated cytokine-containing solution (37°C in serum-free medium with 1% ITS-X) was then added to the melted 1% agarose to produce a final concentration of 0.5% agarose and a 10 *μ*g/mL cytokine solution. A negative control agarose solution was prepared by using reconstitution buffer for each cytokine. For each condition, 10 independent drops of 5 *μ*L (maintained at 37°C) were pipetted onto a sterile 30 mm diameter glass coverslip previously placed into a 35 mm cell culture Petri dish. The dish was then cooled for 10 min at 4°C to allow the agarose spot to solidify. The petri dishes were then gently filled with 2 mL of serum-free medium with 1% ITS-X and placed in an incubator (37°C in 5% CO_2_) for 10 min. Then, OE-MSCs cultured in serum-containing medium were trypsinised and resuspended in the serum-free medium. Every dish containing the agarose spots was seeded with 2 × 10^5^ cells and then incubated for 24 h (37°C in 5% CO_2_). The number of cells invading underneath the agarose spot was manually counted using standard phase contrast light microscopy. Imaging was performed using an inverted phase microscope (TE300, Nikon) with a 10x objective, and images were acquired using an Orca-ER CCD camera (Hamamatsu Photonics) and Axiovision software (Zeiss). The total number of invading cells per agarose spot was counted, and the reported values are the mean number of cells per spot of at least three independent experiments.

### 2.11. Statistics

All data are presented as mean ± SEM. Data were analyzed using SPSS/PC + statistics 11.0 software (SPSS Inc., Chicago, IL). Kruskal-Wallis, ANOVA, and Mann–Whitney *U* tests were used appropriately to detect any significant difference, as indicated in each figure legend. The minimal threshold for significance was set at *p* < 0.05.

## 3. Results

### 3.1. Human OE-MSCs Migrate through an In Vitro BBB Model

**Figure 1:**
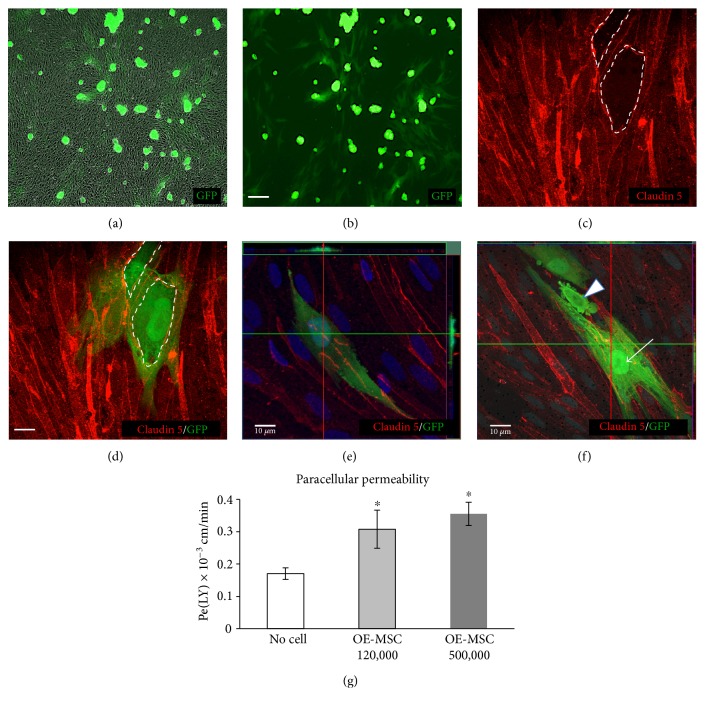
Migration of human olfactory stem cells through the blood-brain barrier (BBB). (a–f) GFP-positive human OE-MSCs were seeded at two densities (120,000 and 500,000 cells per well) on a BBB model, and their diapedesis was observed 24 hours later. Phase contrast (a) and fluorescent (b) photomicrographs displaying globular OE-MSCs adhering on the external surface of the endothelial cell layer and attached OE-MSCs with processes indicating their localization on the membrane and under the BBB. Scale bar: 100 *μ*m. (c) Anti-Claudin 5 antibody identifies endothelial tight junctions (red). (d) Immunostaining reveals openings in the BBB (white dotted lines) and the integration of two OE-MSCs under the BBB. Scale bar: 10 *μ*m. (e, f) Focus on migrating cells. (e) The sequential plane bars (spaced 0.4 *μ*m), on the top and the right, indicate that the migrating cell (green) is located under the Claudin 5-stained endothelial cells (red). (f) Focus on a GFP-positive OE-MSC during transmigration (white arrowhead) and on another one installed under the BBB (white arrow). (g) One day after seeding, BBB permeability was measured by a Lucifer yellow test. The fluorometric measurement of Lucifer yellow indicates that stem cell increases BBB permeability at both cell densities (*n* = 4). ^∗^*p* < 0.05.

To visualize the diapedesis phenomenon, we plated GFP-positive OE-MSCs on an in vitro model of blood-brain barrier. At low magnification and using phase contrast (Figure 1(a)) and epifluorescence (Figures 1(a) and 1(b)), we observed apparently undifferentiated stem cells adhering onto the apical surface of the endothelial cell layer as well as differentiating-looking cells, spreading out on the membrane of the insert and underneath the basal side of the BBB. To cross the barrier, the stem cells can either go through one endothelial cell (transcellular) or between endothelial cells (paracellular). For the latter, they possibly perforate the layer, in various places, to make their way to the other side, as observed in Figures 1(c) and 1(d). Using an anti-Claudin 5 antibody to label tight junctions, we revealed breaches in the BBB, close to two GFP+ stem cells. To ascertain barrier crossing, we used the orthogonal projection bars of a confocal microscope, as shown in Figure 1(e) (top and right side bars): the migrating cell (green) is clearly located under the Claudin-positive endothelial cell layer (red).  Figure 1(f) displays a GFP-positive globular OE-MSC during migration (white arrowhead) and an attached OE-MSC under the BBB (white arrow).

The observed perforation of the barrier led us to measure its permeability. We cocultivated stem and endothelial cells during 24 hours, and using a 1-hour long Lucifer yellow test, we observed a significant increase in barrier permeability, for both cell densities: *p* = 0.042 at 120,000 cells per well and *p* = 0.020 at 500,000 cells per well (Figure 1(g)).

### 3.2. OE-MSCs Secrete Proteins Involved in Homing and Transmigration

**Figure 2:**
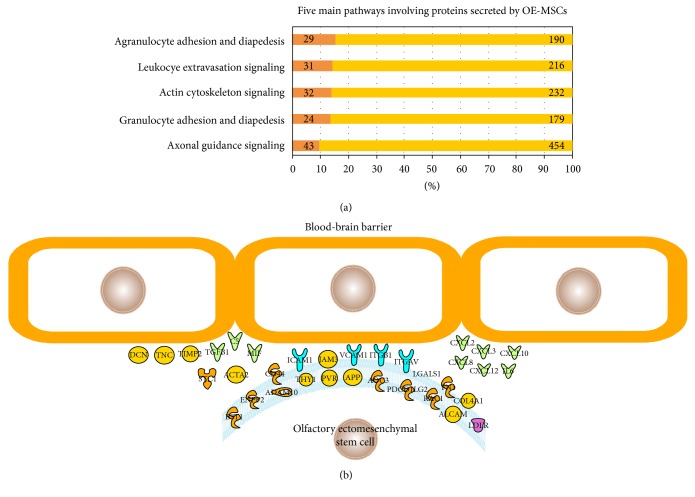
Bioinformatic analysis of the OE-MSC secretome. The 629 proteins secreted by OE-MSCs identified by mass spectrometry were analyzed using the Ingenuity Pathway software. (a) List of the five top canonical pathways, ranked by decreasing ratio values (percentage of identified secreted proteins in the pathway over a total of known proteins involved in this pathway). The numbers in the darker boxes indicate the number of OE-MSC-secreted proteins associated with the pathway; the number at the end of the bar represents the sum of known proteins associated with each specific pathway. (b) Schematic view of the 34 OE-MSC-secreted proteins involved in transmigration. The list of secreted proteins associated with cell movement and homing are found in Supplementary Table 1.

In order to identify diapedesis mechanisms, we sought soluble factors secreted by OE-MSCs. It was surmised that some of them are responsible, at least partly, for the transmigration ability of olfactory stem cells. To this end, we analyzed the medium in which the stem cells were cultured for 3 days, called conditioned media. After filtration, purification of the protein fraction, and migration on agarose gel, we identified the proteins present using a mass spectrometer. A total of 629 human proteins were characterized (see Supplementary Table 1). Nearly two out of five (38% = 239/629) of these proteins are associated with cell movement, transmigration, and/or homing. The full list of 629 proteins was analyzed with the Ingenuity software. As indicated in Figure 2(a), the five top canonical pathways are associated with diapedesis/extravasation (3/5), actin cytoskeleton (1/5), and axon guidance (1/5). Ingenuity Pathway Analysis also identified the 34 proteins associated with transmigration, and we propose the diagram in Figure 2(b) to account for possible OE-MSC diapedesis mechanisms.

### 3.3. Lesioned Hippocampi Are Inflamed One Month after Injury

**Figure 3:**
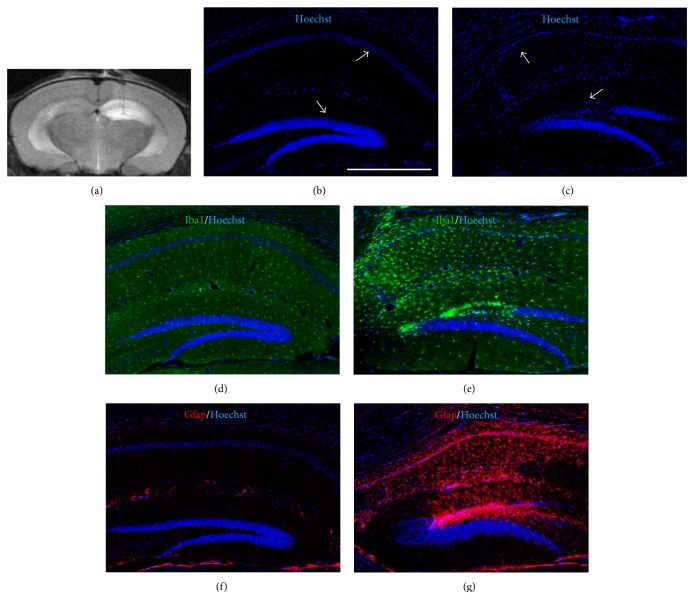
Inflammation in the lesioned hippocampus. (a) MRI assessment of an in vivo lesion, 24 hours after injection of ibotenic acid in the right hippocampus. Example of an axial contiguous T2-weighted image (slice thickness = 500 *μ*m, TEeff = 60 ms, TR = 3000 ms, rare factor = 8; 8 averages). The hypersignal (bright intensity) in the right hippocampus reveals the extent of the injury. Hoechst blue staining on representative coronal brain sections at the dorsal hippocampal level shows the extent of ibotenic acid-induced neuronal death, one month after the lesion (c), in CA1 and dentate gyrus, in comparison with the unlesioned (b) controlateral hippocampus (white arrows). (d–g) Brain sections were immunostained with anti-Iba1 (in green) and anti-Gfap (in red) antibodies. The four-week-lesioned hippocampus displays numerous activated astrocytes and microglia, in comparison with the healthy controlateral hippocampus. Similar images were obtained with at least three brains. Scale bar: 0.5 mm.

We then wanted to evaluate the role of inflammatory processes in OE-MSC selective migration toward lesioned hippocampi [13]. We first used MRI to validate the specificity and efficacy of the lesion in each animal. Twenty-four hours after surgery, we visualized the injected/lesioned area and confirmed that only the targeted hippocampus was affected (Figure 3(a)). Along this line, brain tissue analysis at four weeks postlesion showed a dramatic cell loss in the injected hippocampal cell layers and more particularly in the CA1 and dentate gyrus regions (Figures 3(b) and 3(c)). Additionally, we observed that neuroinflammation markers such as Gfap and Iba1, which, respectively, mark reactive astrocytes and microglia, were strongly and specifically expressed in the lesioned areas (Figures 3(d)–3(g)). Thus, one month after injury, the lesioned hippocampi still displayed inflammation hallmarks, making this model highly suitable for further gene expression analyses.

### 3.4. Lesioned Hippocampi Overexpress Genes Involved in Cell Chemoattraction

**Figure 4:**
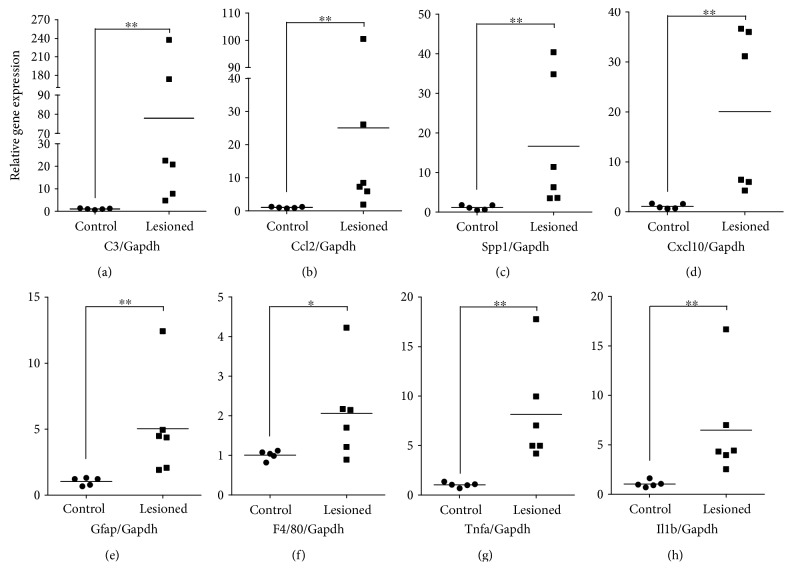
Confirmation of cytokine overexpression in the four-week-lesioned hippocampus by RT-qPCR. Scatterplots showing relative expression of selected cytokine transcripts in four-week-lesioned hippocampi (*n* = 6), in comparison with healthy hippocampi (*n* = 5). (a–d) Overexpression of transcript coding for chemotactic cytokines selected after the microarray experiment (i.e., C3, Ccl2, Spp1, and Cxcl10) was confirmed by RT-qPCR. (e–h) Expression of genes known to be overexpressed in the inflamed brain (i.e., Gfap, F4/80, Tnfa, and Il1b) was also assessed. Relative expression levels were determined according to the ΔΔCt method, the Gapdh gene serving as endogenous control for normalization. Each point corresponds to one mouse. The horizontal bars indicate the mean relative gene expression. (^∗^*p* ≤ 0.05 and ^∗∗^*p* ≤ 0.01, using two-tailed Mann–Whitney *U* test.)

In order to find candidate OE-MSC chemoattractants within lesioned brain tissue, we established the gene expression profiles of control and lesioned hippocampi. Four weeks after the lesion, hippocampi were isolated and processed for pan-genomic microarray analysis (ArrayExpress database, accession number E-MEXP-2682). The data indicated that 114 transcripts with a fold change above 2.5 were upregulated in the lesioned hippocampi. Interestingly, the DAVID Functional Annotation Clustering Tool showed that overexpressed transcripts clustered into functional groups, indicating in particular that 53% of these genes are involved in immune and inflammatory processes. More importantly, among the strongly upregulated transcripts (fold change above 10), four were coding for molecules already known for their chemotactic properties on immune cells and other stem cell types (Table 1). Indeed, transcripts for *C3* and *Spp1*, two members of the cytokine family, as well as those for the *Ccl2* and *Cxcl10* chemokines appeared as highly interesting hits. Beside those four candidates, other chemotactic cytokine transcripts (*Ccl4*, *Ccl5*, *Ccl7*, *Ccl12*, *Ccl19*, *Cxcl1*, and *Cxcl16*) were also significantly overexpressed. On the contrary, other genes did not display significant changes though they code for cytokines or growth factors with a chemoattractant role on different types of stem cells, such as *Cxcl12/Sdf1*, stem cell factor (*SCF*), *Igf1*, *Hgf*, *Pdgfa*, and *Vegfa*. In the subsequent experiments, we decided to focus on the four most overexpressed chemotactic cytokines. To confirm gene expression data from our microarray analysis, we assessed the expression level of *C3*, *Spp1*, *Ccl2*, and *Cxcl10* by RT-qPCR (Figure 4). Here again, the selected cytokines were significantly overexpressed (*p* < 0.01 for each) in lesioned hippocampi (*n* = 6) when compared to nonlesioned hippocampi (*n* = 5) (Figures 4(a)–4(d)). Noticeably, gene expression analysis on another set of genes involved in brain inflammation—that is, *Gfap*, *F4/80*, *Tnfa*, and *Il1b*—confirmed the inflammatory state of the injured hippocampi, one month after lesion (Figures 4(e)–4(h)).

### 3.5. CCL2 and SPP1 Stimulate OE-MSC Migration In Vitro

**Figure 5:**
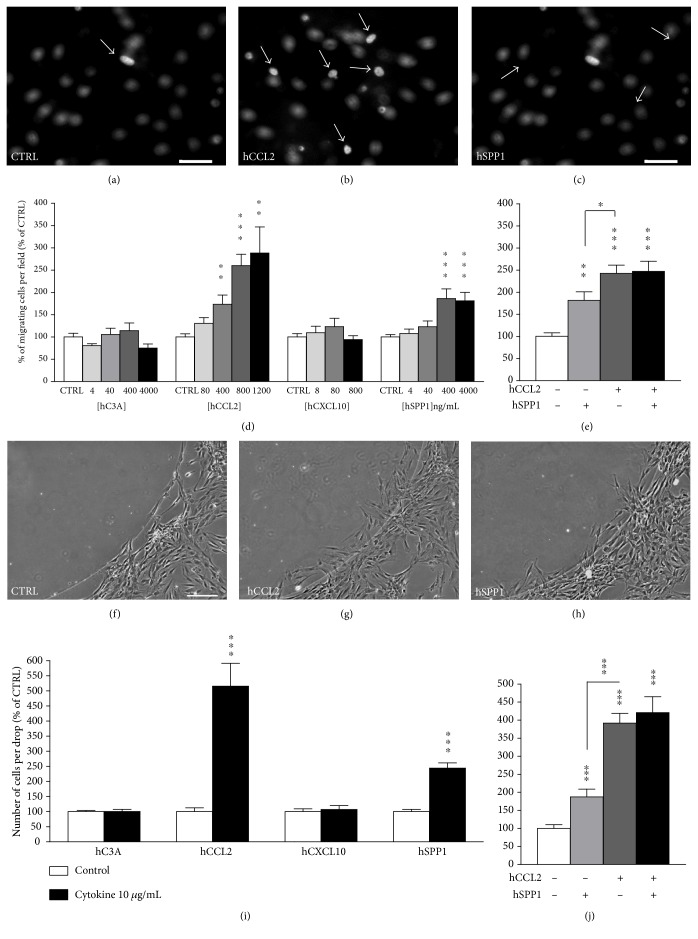
Among selected overexpressed cytokines, only CCL2 and SPP1 stimulate OE-MSC migration. Effects of human recombinant cytokines (i.e., hC3A, hCCL2, hSPP1, and hCXCL10) on human OE-MSC migration were assessed using modified Boyden chambers (a–e) and agarose spot assay (f–j). (a–c) Representative images of membrane filter fields from modified Boyden chambers display Hoechst-stained nuclei of migrating human OE-MSCs, 12 hours after the addition of buffer or cytokines (1,000 ng/mL) in the lower chamber. Under the 40x objective, the migrating stem cells attached to the lower side of the membrane (white arrows) were easily distinguishable from nonmigrating cells (unfocused nuclei). Scale bar: 50 *μ*m. Mean percentage of migrating OE-MSCs per field in response to cytokines at increasing concentrations (d) or to a cocktail of the two active cytokines at the concentration showing maximum efficacy (1,000 ng/mL) (e). (f–h) Representative images of an area of jellified agarose drop (upper left zone) containing buffer or cytokines at 10 *μ*g/mL. Twenty-four hours after plating, OE-MSCs were observed around the drop and human OE-MSCs penetrated into the drop in the presence of active cytokines (hCCL2, hSPP1). Scale bar: 200 *μ*m. Mean number of migrating human OE-MSCs in the drop area in response to buffer or cytokines at 10 *μ*g/mL (i) or a cocktail of the two active cytokines used at the same concentration (j). Values reported are the mean (±SEM) of at least three independent experiments performed in triplicate for modified Boyden chambers and in ten replicate drops for agarose spot assays. (^∗∗^*p* ≤ 0.01 and ^∗∗∗^*p* ≤ 0.001, using Student's *t*-test.)

**Figure 6:**
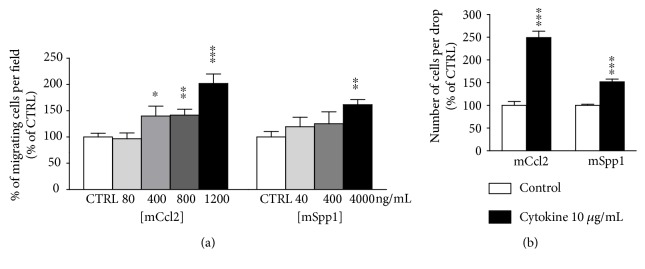
Mouse recombinant mCcl2 and mSpp1 cytokines also stimulate human OE-MSC migration. Effects of mouse recombinant cytokines (i.e., mCcl2 and mSpp1) on human OE-MSCs were assessed using modified Boyden chambers (a) and agarose spot assays (b). Mean percentage of migrating human OE-MSCs per field in response to buffer or cytokines at increasing concentrations, 12 hours after the start of the experiment (a). Mean number of migrating human OE-MSCs per drop in response to buffer or cytokines at 10 *μ*g/mL, 24 hours after the start of the experiment (b). Values reported are the mean (±SEM) of at least three independent experiments performed in triplicate for modified Boyden chambers and in ten replicate drops for agarose spot assays. (^∗^*p* ≤ 0.05, ^∗∗^*p* ≤ 0.01, and ^∗∗∗^*p* ≤ 0.001, using Student's *t*-test.)

Our converging data led us to evaluate the effects of the human recombinant cytokines C3A, CCL2, SPP1, and CXCL10 on human OE-MSC migration. To this end, we used modified Boyden chambers and agarose spot assays to assess the chemotattractant potential of these molecules on OE-MSCs. Interestingly, only CCL2 and SPP1 stimulated OE-MSC migration in modified Boyden chambers. Indeed, we observed a significantly increased number of migrating cells in response to either of these two cytokines as early as 12 hours after treatment (Figures 5(a)–5(d)). Noticeably, our results showed a dose-dependent effect, starting to be significant when the concentration reached 400 ng/mL for both CCL2 (*p* < 0.01) and SPP1 (*p* < 0.001) (Figure 5(d)), and no cumulative effect when both cytokines were used (Figure 5(e)). The total number of cells was unchanged under these conditions, thus excluding an artifactual effect due to OE-MSC increased proliferation. Of note, these results have been consistently reproduced on OE-MSCs derived from three additional individuals (data not shown). Similarly, we observed that only CCL2 and SPP1 significantly stimulate the migration of OE-MSCs under jellified agarose drops containing the cytokines at 10 *μ*g/mL (*p* < 0.001 for both), after 24 hours of migration (Figures 5(f)–5(i)). Here again, no additive effect was observed when the two cytokines were combined (Figure 5(j)). Altogether, our results indicate that CCL2 is more potent than SPP1 in the modified Boyden chamber assay (242.7 ± 18.6% for hCCL2 and 181.4 ± 19.5% for hSPP1; *p* < 0.05) as well as in the agarose spot assay (391.5 ± 27.2% for hCCL2 and 187.1 ± 21.7% for hSPP1; *p* < 0.001), when comparing the cytokines at the concentration with maximum efficacy. With these data in hand, we hypothesized that the same cytokines might play a role in the migration of OE-MSC into lesioned mouse hippocampi [13]. We used a similar strategy with the mouse recombinant Ccl2 and Spp1 cytokines. Similarly to human CCL2 in the modified Boyden chamber assay, mouse Ccl2 stimulated OE-MSC migration, starting from the concentration of 400 ng/mL (*p* < 0.05) (Figure 6(a)). Surprisingly, a higher concentration of mouse Spp1 (4,000 ng/mL) was required to observe an effect (*p* < 0.01) (Figure 6(a)). Finally, the agarose spot assay confirmed that Ccl2 and Spp1 were able to stimulate OE-MSC migration (*p* < 0.001 for both) with Ccl2 being clearly more potent than Spp1 (Figure 6(b)). Altogether, our results validated that CCL2 and SPP1 are two candidate molecules that could participate in the directed migration of OE-MSCs toward a specific lesioned area.

### 3.6. CCL2 Exerts a Chemotactic Effect on OE-MSCs In Vitro

**Figure 7:**
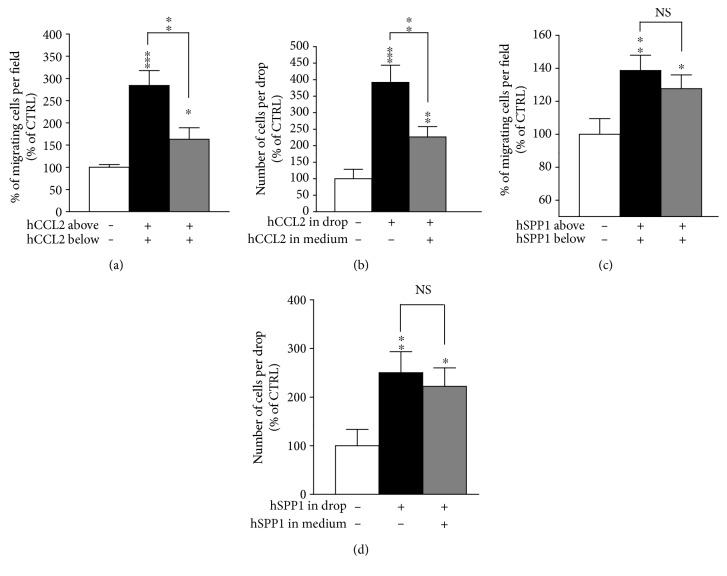
CCL2 stimulates both chemokinesis and chemotaxis while SPP1 mainly acts on chemokinesis. Random cell motility (chemokinesis) and gradient-dependent cell migration (chemotaxis) of human OE-MSCs in response to human recombinant cytokines hCCL2 and hSPP1 were assessed using modified Boyden chambers (a, c) and agarose spot assays (b, d). (a, c) Mean percentage of migrating human OE-MSCs per field, 12 hours later, in response to buffer or cytokines placed either in the lower well (below) or in both the upper and the lower wells (above and below). (b, d) Mean number of migrating human OE-MSCs per drop, 24 hours later, in response to buffer or cytokines placed either in the agarose drop or in both the medium and the drop. Values reported are the mean (±SEM) of at least three independent experiments performed in triplicate for modified Boyden chambers and in ten replicate drops for agarose spot assays. (^∗^*p* ≤ 0.05, ^∗∗^*p* ≤ 0.01, and ^∗∗∗^*p* ≤ 0.001, using Student's *t*-test.)

In order to further elucidate CCL2 and SPP1 mechanisms of action on OE-MSC migration, we then evaluated the relative contribution of random cell motility (chemokinesis) and gradient-dependent cell migration (chemotaxis) of human OE-MSCs in response to CCL2 and SPP1. To distinguish chemotaxis from chemokinesis in modified Boyden chambers, we compared the effect of the cytokines placed either in the lower well only or in both the upper and the lower compartments of the chamber, in order to disrupt the gradient. Similarly, in agarose spot assays, the cytokines were also added in the medium around the drop, in order to disrupt the cytokine gradient. Interestingly, we observed a significant reduction of the CCL2-dependent stimulation in gradient-disrupted conditions (*p* < 0.01 for both assays) (Figures 7(a) and 7(b)) suggesting a chemotactic effect of CCL2 on OE-MSCs. Yet, OE-MSC migration still increased in the absence of a CCL2 gradient (*p* < 0.05 in modified Boyden chambers and *p* < 0.01 in agarose spot assay), thus indicating that CCL2 stimulates a combination of both chemokinesis and chemotaxis. Importantly, no significant difference was observed when the SPP1 gradient was disrupted (Figures 7(c) and 7(d)), indicating that SPP1 only acts on human OE-MSC chemokinesis.

### 3.7. Human OE-MSCs Express CCR1 and CCR10 but Not CCR2

**Figure 8:**
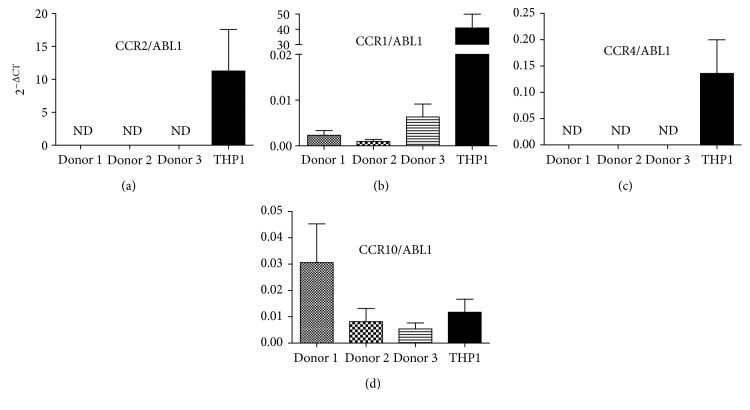
Expression of CCR receptors in human OE-MSCs suggests CCL2 acts through unsuspected pathways. (a–d) mRNA expression of C-C chemokine receptors (CCRs) that can potentially bind CCL2 (i.e., CCR2, CCR1, CCR4, and CCR10), normalized to the *ABELSON* housekeeping gene (ABL1). Values reported are the mean (±SEM) of at least three independent experiments performed in duplicate. Expression level of each gene is represented by 2^−∆CT^ where ∆CT = CT (gene X) − CT (ABL1). Human OE-MSCs from three donors (donors 1, 2, and 3) were analyzed. The acute monocytic leukemia cell line THP1 was used as positive control. ND: not detected.

In view of our results, we decided to assess the expression in OE-MSCs of the cognate receptor of CCL2 and CCR2. To this end, we designed RT-qPCR probes (Supplementary Table 2) against the two isoforms of *CCR2* (*CCR2A* and *CCR2B*). Surprisingly, *CCR2* mRNA was undetectable in OE-MSCs from all three tested donors (Figure 8(a)). Of note, the detection of *CCR2* mRNA in an acute monocytic leukemia cell line (THP1) known to express *CCR2* demonstrated the validity of our system of analysis. To elucidate an alternative mechanism of action bypassing the classic CCL2/CCR2 interaction, we measured the expression of C-C chemokine receptors (CCRs) that can potentially bind CCL2, including CCR1, CCR4, and CCR10. Both *CCR1* and *CCR10* were expressed in human OE-MSCs, thus offering two alternative ways of action for CCL2 on OE-MSCs.

## 4. Discussion

In a previous study, we demonstrated that a directed migration of human OE-MSCs into the lesioned hippocampus accompanied the functional recovery observed after their transplantation in an amnesic mouse model. We reported the ability of OE-MSCs to migrate in vivo when the transplantation was performed at a distance from the injury site [13]. However, molecular mechanisms regulating the recruitment and the homing of OE-MSCs into injured brain areas had not yet been investigated and gaining insight into these mechanisms could improve the therapeutic potential of grafted OE-MSCs. We provide here the first evidence that human OE-MSCs secrete key proteins that allow them to cross the blood-brain barrier and are attracted by components of the innate immune system overexpressed in the lesioned hippocampus. We identified CCL2 as a chemokine able to stimulate the gradient-dependent migration of human OE-MSCs whereas the SPP1 cytokine enhances their random motility.

To date, no study has ever reported the migration of OE-MSCs through the blood-brain barrier. However, according to our prior work, OE-MSCs belong to the subgroup of ectomesenchymal cells [11], they are members of the large family of mesenchymal cells. Interestingly, several studies reported bone marrow mesenchymal stem cells passing through the BBB [36–38].

Throughout the current manuscript, we use the word “diapedesis” to evoke the passage of OE-MSCs through the BBB model. This term often refers to the mechanism by which a cell (usually a leukocyte) finds its way between the endothelial cells of a blood capillary. There is a second possibility for crossing the BBB: the transcellular way which allows a molecule or a cell to cross the cytoplasm of an endothelial cell to join either the lumen of a blood vessel or an organ [39]. The data presented here argue in favor of perforations of the endothelial layer at the close junctions between endothelial cells. However, the parallel existence of transcytosis cannot be ruled out. To resolve this issue in future studies, we could not only use video microscopy and electron microscopy but also study the expression of proteins involved in transcellular migration (JAM-A, PECAM-1, VCAM-1, ICAM-1, etc.) [40].

The present study identified 629 OE-MSC-secreted proteins, more than twice the number (274) of secreted proteins reported by a research team studying the same adult stem cell subtype [41]. Both datasets contained 119 common proteins while 510 were uniquely uncovered in our study. The sensitivity of the mass spectrometer used in our experiments may explain such a discrepancy. Nonetheless, both studies underline the importance of cell migration. Indeed, using Swiss-prot functional annotation, the previous study observed that the largest number of secreted proteins was associated to the cell growth and migration pathway [41]. Similarly, we report here that 239 out of 629 OE-MSC-secreted proteins are associated to cell movement, migration, and/or homing.

Neuroinflammation is a common denominator in damaged cerebral areas, and cytokines associated with neuroinflammation are known to be involved in the homing mechanisms of different types of immune, tumor, and stem cells [42]. Thus, deciphering the inflammatory processes in damaged areas is a prerequisite to understanding human OE-MSC homing mechanisms. Histological analysis of 4-week-lesioned hippocampi revealed a strong reactivity of astrocytes and microglia, two major components of the brain innate immunity [43]. As expected, lesioned areas displayed the gene expression profile of an inflamed structure and a majority of overexpressed genes directly participate in immune and inflammatory processes. In an attempt to elucidate the signals involved in OE-MSC homing, we identified four highly overexpressed transcripts that code for two cytokines (i.e., *C3* and *Spp1*) and two chemokines (i.e., *Ccl2* and *Cxcl10*) known for their chemotactic properties on immune cells or on various stem cell types.

The first candidate, the complement component C3, is the most overexpressed transcript of the microarray. The complement cascade is activated in most neurological diseases, including acute or chronic brain insults [44]. Complement activation from classical, alternative, and lectin pathways leads to production of the anaphylatoxin C3a, a small polypeptide released from the precursor protein C3 by C3 convertases. C3a anaphylatoxin is involved in inflammatory processes following focal cerebral ischemia [45] and has been described as a chemotactic factor for mesenchymal stem cells in vitro [46]. Similarly, SPP1, also called osteopontin, exhibits strong chemoattractive and proinflammatory properties [47], is upregulated in injured brain areas [48, 49], and promotes the migration of various cell types, including astrocytes, mesenchymal stem cells, or neuroblasts from the subventricular zone [48, 50–53]. CXCL10, a member of the CXC chemokine family, can also attract bone marrow-derived mesenchymal stem cells (BM-MSCs) [54], and CXCL10-expressing cells appear in the inflamed central nervous system after a trauma or in neurodegenerative diseases [55]. Among the four candidates, CCL2 is the most studied factor in terms of stem cell homing into damaged areas: it displays chemoattractive properties on numerous stem cell types, including neural crest-derived stem cells [56], neural stem cells [28, 29, 57, 58], and BM-MSCs [59, 60].

When we tested the effects of these 4 overexpressed cytokines on OE-MSC migration, the data from agarose spot assays perfectly corroborated the findings obtained with modified Boyden chambers. We demonstrated that human recombinant SPP1 significantly stimulated human OE-MSC migration from a concentration range of 40–400 ng/mL. These results are in agreement with a previous study showing that SPP1 increases the migration of BM-MSCs at concentrations of 500 ng/mL and above [51]. However, other studies reported an effect of SPP1 with concentrations ranging from 10 to 20 *μ*g/mL on astrocytes [48], mesenchymal stem cells [50], and neuroblasts [52, 53], in modified Boyden chambers. This comparison highlights the high sensitivity of human OE-MSCs to SPP1. This cytokine is known to interact with different cell surface receptors [47] such as integrin β1 [51, 53] and integrin *α*v [49] via an arginine-glycine-aspartate (RGD) domain or with CD44 via an RGD-independent mechanism [50]. In a previous study [11], we demonstrated that human OE-MSCs strongly express integrin *β*1 (CD29), integrin *α*v (CD51), and CD44 at their surface.

In regard to the CCL2 chemokine, we demonstrated a significant effect of human recombinant CCL2 on human OE-MSC migration, with concentrations ranging from 80 to 400 ng/mL. These results are in agreement with previous studies showing that CCL2 affects neuroblasts, at 500 ng/mL [29] or on BM-MSCs from 75 ng/mL, in modified Boyden chambers [61]. However, numerous in vitro studies reported a CCL2 effect at lower concentrations (150 pg/mL to 10 ng/mL) on neuroblasts and mesenchymal stem cells [28, 56, 57, 59, 62, 63]. In most cases, the studied cells expressed the CCR2 receptor. Surprisingly, we did not detect the two cognate CCL2 receptors generated by alternative splicing, namely, *CCR2A* and *CCR2B*, in human OE-MSCs from three different donors. A different picture emerges from studies on BM-MSCs, a stem cell type closely related to OE-MSCs [11]: on these cells, the expression of chemokine receptors and particularly CCR2 varies according to culture conditions and assessment techniques [64–67]. CCR2 expression is stimulated by preincubating the BM-MSCs with inflammatory cytokines [65]. However, pretreating OE-MSCs with the proinflammatory cytokine TNFa or the CCL2 ligand did not induce CCR2 expression in OE-MSCs (data not shown). These observations suggest that CCL2 stimulates OE-MSC migration via a CCR2-independent pathway. Chemokines bind to a large family of receptors with various binding affinities [68, 69]. For example, although CCR2 is the receptor with the highest affinity, CCL2 can bind to lower affinity receptor, such as CCR1 [68, 70] and CCR4 [71–73], or via unknown mechanisms [74, 75]. In the current study, using RT-qPCR, we observed a weak expression of *CCR1* while *CCR4* transcripts were undetectable. Unpublished microarray data obtained on OE-MSCs indicate that *CCR10* is the most highly expressed C-C chemokine receptors (CCR) in these cells. Here, we confirmed its expression in OE-MSCs using RT-qPCR. Although CCR10 is mainly known to bind CCL27 and CCL28 with high affinity [76], CCL2 may nonetheless bind to and activate CCR10 in OE-MSCs, through a yet unknown pathway.

When comparing the effects of CCL2 and SPP1 on OE-MSC migration, we observed that CCL2 is more potent and is the prime candidate to explain OE-MSC homing into the lesioned hippocampus. It is now established that a molecule can affect cell migration by stimulating either random motility (i.e., chemokinesis) or concentration-dependent-directed migration (i.e., chemotaxis) or a combination of both [77]. The CCL2 chemokine is efficient on OE-MSC migration in the presence of a chemokine gradient, suggesting that CCL2 effects are mainly chemotactic, whereas SPP1 induces similar responses in the presence or absence of a gradient, demonstrating that its effects are mainly chemokinetic. Moreover, at the concentration showing the maximum efficacy for the two cytokines, CCL2 is more potent than SPP1 and a cocktail of both cytokines does not increase CCL2 effects. The concentration required to stimulate the in vitro migration of human OE-MSCs suggests that the potential effect of these cytokines in vivo may be restricted to major physiopathological inflamed conditions. However, we cannot exclude that OE-MSCs express higher levels or new chemokine receptors when they are grafted into the brain. Alternatively, cocktails of several other chemokines, expressed at lower level in the inflamed hippocampus, may also exert chemotactic effects.

In a previous study, we successfully demonstrated the therapeutic potential of human OE-MSCs grafted in a murine model of amnesia [13]. Therefore, we decided to assess the chemoattractive potential of human and murine cytokines. In regard to CCL2, comparing the amino-acid sequences between human and murine CCL2 indicates a 55% identity and an 81% similarity [78]. Predictably, we demonstrated here that mouse and human recombinant CCL2 induce similar effects on human OE-MSC migration. This finding is in line with a prior study showing that human and murine cytokines are equiactive on human monocytes [79]. Concerning SPP1, comparing the amino-acid sequences between human and murine SPP1 indicates a 64% identity and an 81% similarity [80]. Like CCL2, we show here that mouse and human recombinant SPP1 stimulate human OE-MSC migration.

## 5. Conclusion

In summary, we bring the first evidence that human OE-MSCs express transmigration-associated proteins, cross the blood-brain barrier, and respond to cytokines overexpressed in lesioned hippocampi, such as CCL2 and SPP1. We also discovered which cerebral constituents may attract OE-MSCs and be responsible, at least partially, for their homing into sites of cerebral injuries. These new data might be proven crucial for improving the therapeutic potential of OE-MSCs with a view to repairing the pathological or traumatized human brain.

## Supplementary Material

The information of supplementary materials are as follows: Supplementary table 1. List of proteins secreted by OE-MSCs. Supplementary table 2: Mouse and human target and control probes used in RT-qPCR.

## Figures and Tables

**Table 1:** Dysregulated transcripts with a fold change above 10 in the lesioned hippocampus, in comparison with the unlesioned hippocampus, one month after the lesion.

Gene symbol	Gene/protein name	Fold change
***C3***	**Complement component 3**	**162.5**
*Lyz1*	Lysozyme 1	31.4
*Mmp3*	Matrix metalloproteinase 3	27.1
***Ccl2***	**Chemokine (C-C motif) ligand 2**	**24.2**
*Lyz2*	Lysozyme 2	23.7
*Clec7a*	C-type lectin domain family 7, member a	23.5
*Tnfaip2*	Tumor necrosis factor, alpha-induced protein 2	14.1
*Tgm1*	Transglutaminase 1, K polypeptide	13.4
***Spp1***	**Secreted phosphoprotein 1**	**12.1**
*Timp1*	Tissue inhibitor of metalloproteinase 1	11.9
***Cxcl10***	**Chemokine (C-X-C motif) ligand 10**	**11.7**
*Bcl3*	B-cell leukemia/lymphoma 3	11.7
*Chi3l1*	Chitinase 3-like 1	10.8

Microarray data indicate that 13 genes are overexpressed in lesioned hippocampi with a fold change above 10, one month after the lesion. All are known to be involved in immune and inflammatory processes and in tissue remodeling. Among these genes, 4 codes for cytokines with demonstrated chemotactic properties: *C3*, *Ccl2*, *Spp1*, and *Cxcl10* (bold).
