# Supplementary material for: From Blood to Lesioned Brain: An In Vitro Study on Migration Mechanisms of Human Nasal Olfactory Stem Cells
Source: Stem Cells Int. 2017 Jun 18;2017:1478606. doi: 10.1155/2017/1478606 (PMC5494110; doi:10.1155/2017/1478606)
Supplement: Supplementary file 1 — The information of supplementary materials are as follows: Supplementary table 1. List of proteins secreted by OE-MSCs. Supplementary table 2: Mouse and human target and control probes used in RT-qPCR. [file 1478606.f1.docx]

**Supplementary table 1**. List of proteins secreted by OE-MSCs

| **A1BG** |
| --- |
| **A2M** |
| **ABCA10** |
| **ABHD14B** |
| **ACTA2** |
| **ACTN1** |
| **ACTN2** |
| **ACTN3** |
| **ACTN4** |
| **ACTR2** |
| **ADAM10** |
| **ADAM19** |
| **ADAM9** |
| **ADAMTS1** |
| **ADAMTS13** |
| **ADAMTS2** |
| **ADAMTS7** |
| **ADAMTSL1** |
| **ADPRHL2** |
| **AEBP1** |
| **AFM** |
| **AFP** |
| **AGA** |
| **AGRN** |
| **AHCY** |
| **AKR1A1** |
| **AKR1B1** |
| **ALCAM** |
| **ALDOA** |
| **ALDOB** |
| **ALDOC** |
| **AMBP** |
| **ANGPTL4** |
| **ANKRD30A** |
| **ANPEP** |
| **AOC3** |
| **APLP2** |
| **APOA1** |
| **APOB** |
| **APOH** |
| **APOM** |
| **APP** |
| **ARF1** |
| **ARHGAP35** |
| **ARHGEF18** |
| **ARPC1B** |
| **ARSA** |
| **ASAH1** |
| **ASTN2** |
| **ATP5B** |
| **ATRN** |
| **B2M** |
| **B3GAT3** |
| **B3GNT9** |
| **B4GALT1** |
| **B4GALT4** |
| **B4GALT5** |
| **B4GAT1** |
| **BASP1** |
| **BAX** |
| **BBS7** |
| **BCAT1** |
| **BGN** |
| **BPTF** |
| **BRWD3** |
| **BST1** |
| **BTD** |
| **C10orf11** |
| **C1QTNF1** |
| **C1QTNF3** |
| **C1QTNF5** |
| **C1R** |
| **C1RL** |
| **C1S** |
| **C2** |
| **C3P1** |
| **C4A/C4B** |
| **C5** |
| **C7** |
| **C9** |
| **CACNA2D1** |
| **CACNG6** |
| **CALML5** |
| **CALR** |
| **CALU** |
| **CAMSAP2** |
| **CAND1** |
| **CANT1** |
| **CAP1** |
| **CAPG** |
| **CAPZA1** |
| **CAPZA2** |
| **CAPZB** |
| **CASP8** |
| **CBR1** |
| **CBR3** |
| **CCDC80** |
| **CCDC88C** |
| **CCKAR** |
| **CD248** |
| **CD44** |
| **CD59** |
| **CD63** |
| **CD9** |
| **CDH13** |
| **CDH2** |
| **CDK5RAP2** |
| **CENPF** |
| **CFAP54** |
| **CFH** |
| **CFL1** |
| **CFL2** |
| **CHD1L** |
| **CHD9** |
| **CHID1** |
| **CHRDL1** |
| **CHST14** |
| **CHST3** |
| **CKMT2** |
| **CLEC11A** |
| **CLEC3B** |
| **CLIC1** |
| **CLIC4** |
| **CLN5** |
| **CLSTN1** |
| **CLSTN2** |
| **CLSTN3** |
| **CLU** |
| **CNPY2** |
| **CNTN1** |
| **COL10A1** |
| **COL11A1** |
| **COL11A2** |
| **COL12A1** |
| **COL14A1** |
| **COL15A1** |
| **COL16A1** |
| **COL18A1** |
| **COL2A1** |
| **COL3A1** |
| **COL4A1** |
| **COL4A2** |
| **COL4A3** |
| **COL4A5** |
| **COL5A1** |
| **COL5A2** |
| **COL6A1** |
| **COL6A2** |
| **COL6A3** |
| **COL9A1** |
| **COMP** |
| **COPE** |
| **CORO1C** |
| **COTL1** |
| **CP** |
| **CPE** |
| **CPQ** |
| **CPXM2** |
| **CRABP2** |
| **CRIM1** |
| **CRK** |
| **CSPG4** |
| **CSRP1** |
| **CST3** |
| **CSTB** |
| **CTBS** |
| **CTF1** |
| **CTGF** |
| **CTSA** |
| **CTSB** |
| **CTSL** |
| **CTSZ** |
| **CTTNBP2** |
| **CUTA** |
| **CXCL1** |
| **CXCL10** |
| **CXCL12** |
| **CXCL2** |
| **CXCL3** |
| **CXCL8** |
| **CYCS** |
| **CYR61** |
| **DAG1** |
| **DBT** |
| **DCN** |
| **DDAH1** |
| **DDAH2** |
| **DDT** |
| **DES** |
| **DIAPH1** |
| **DKK3** |
| **DLD** |
| **DNAJB11** |
| **DPP7** |
| **DPYSL2** |
| **DPYSL3** |
| **DST** |
| **DSTN** |
| **ECM2** |
| **EDIL3** |
| **EEF2** |
| **EFEMP1** |
| **EFEMP2** |
| **EFNB1** |
| **EHD2** |
| **EIF4A2** |
| **EMILIN1** |
| **ENO2** |
| **ENO3** |
| **ENOPH1** |
| **ENPP2** |
| **EPDR1** |
| **EPOR** |
| **ERAP1** |
| **ERAP2** |
| **ERO1A** |
| **ERO1B** |
| **ERP44** |
| **EXT1** |
| **EXT2** |
| **EZR** |
| **F13A1** |
| **F5** |
| **F9** |
| **FAM3C** |
| **FAM49B** |
| **FAM83C** |
| **FAP** |
| **FBLN1** |
| **FBLN2** |
| **FBLN5** |
| **FBLN7** |
| **FBN1** |
| **FBN2** |
| **FKBP1A** |
| **FKBP9** |
| **FLNA** |
| **FLNC** |
| **FN1** |
| **FSCN1** |
| **FST** |
| **FSTL1** |
| **FSTL3** |
| **FTH1** |
| **FUCA1** |
| **GAA** |
| **GALNT2** |
| **GALNT5** |
| **GALNT6** |
| **GANAB** |
| **GBE1** |
| **GC** |
| **GGH** |
| **GLA** |
| **GLG1** |
| **GLIPR2** |
| **GM2A** |
| **GMFB** |
| **GNPTG** |
| **GNS** |
| **GOT2** |
| **GPC1** |
| **GPI** |
| **GREM1** |
| **GSN** |
| **GSTO1** |
| **GSTP1** |
| **HBE1** |
| **HEBP2** |
| **HEXA** |
| **HEXB** |
| **HGFAC** |
| **HLA-A** |
| **HLA-B** |
| **HLA-C** |
| **HLA-G** |
| **HNRNPA1** |
| **HNRNPD** |
| **HOOK3** |
| **HPR** |
| **HSP90AA2P** |
| **HSP90AB2P** |
| **HSP90B1** |
| **HSPA13** |
| **HSPA2** |
| **HSPA4** |
| **HSPG2** |
| **HTRA1** |
| **ICA1L** |
| **ICAM1** |
| **ICOSLG** |
| **IDH1** |
| **IGFBP2** |
| **IGFBP4** |
| **IGFBP6** |
| **IGFBP7** |
| **IGLL1/IGLL5** |
| **IL36G** |
| **IL6** |
| **IMPA1** |
| **INA** |
| **INHBA** |
| **INPP5F** |
| **IPO5** |
| **IQGAP1** |
| **ITGA3** |
| **ITGA5** |
| **ITGAV** |
| **ITGB1** |
| **ITGBL1** |
| **ITIH1** |
| **ITIH2** |
| **ITIH5** |
| **JAG1** |
| **JAG2** |
| **JAM3** |
| **KNG1** |
| **KRT18** |
| **KRT26** |
| **KRT33B** |
| **KRT34** |
| **KRT36** |
| **KRT74** |
| **KRT85** |
| **KRT86** |
| **LAMB2** |
| **LAMB4** |
| **LAMC1** |
| **LARP6** |
| **LDHA** |
| **LDHAL6A** |
| **LDHB** |
| **LDLR** |
| **LGALS1** |
| **LGALS3BP** |
| **LGMN** |
| **LMAN1** |
| **LMAN2** |
| **LNPEP** |
| **LOC10028896** |
| **LOX** |
| **LOXL1** |
| **LOXL3** |
| **LTBP2** |
| **LTBP3** |
| **LTF** |
| **LUM** |
| **LXN** |
| **MAN1A1** |
| **MAN2A1** |
| **MAN2A2** |
| **MAN2B1** |
| **MANBA** |
| **MASP1** |
| **MASTL** |
| **MATN1** |
| **MATN2** |
| **MATN3** |
| **MDH1** |
| **MDH2** |
| **MEGF6** |
| **MET** |
| **MFAP2** |
| **MIF** |
| **MMP10** |
| **MMP11** |
| **MMP2** |
| **MMP7** |
| **MRC2** |
| **MRPS30** |
| **MSN** |
| **MST1** |
| **MT1E** |
| **MT3** |
| **MTCL1** |
| **MTPN** |
| **MTUS2** |
| **MXRA8** |
| **MYH14** |
| **MYL3** |
| **MYL6** |
| **NCSTN** |
| **NECTIN2** |
| **NEFL** |
| **NEFM** |
| **NEO1** |
| **NID1** |
| **NME2** |
| **NME3** |
| **NPC1** |
| **NRP1** |
| **NTN4** |
| **NUCB1** |
| **NUP133** |
| **NUP155** |
| **OR5AC2** |
| **P3H1** |
| **P3H2** |
| **P3H4** |
| **P4HA1** |
| **P4HA2** |
| **P4HB** |
| **PAM** |
| **PAMR1** |
| **PAPPA** |
| **PARK7** |
| **PCDH10** |
| **PCDH7** |
| **PCLO** |
| **PCMT1** |
| **PCOLCE** |
| **PDCD1LG2** |
| **PDCD6IP** |
| **PDGFC** |
| **PDGFRB** |
| **PDIA3** |
| **PDIA6** |
| **PEBP1** |
| **PFN1** |
| **PGAM1** |
| **PGAM2** |
| **PGD** |
| **PGLS** |
| **PGLYRP2** |
| **PGM1** |
| **PGRMC2** |
| **PHOX2B** |
| **PIK3IP1** |
| **PKLR** |
| **PKM** |
| **PLA2G15** |
| **PLAT** |
| **PLEC** |
| **PLG** |
| **PLOD1** |
| **PLOD2** |
| **PLSCR3** |
| **PLTP** |
| **PLXDC2** |
| **PLXNB2** |
| **PODN** |
| **POLR3B** |
| **POSTN** |
| **POTEE** |
| **POTEJ** |
| **POTEKP** |
| **PPIA** |
| **PPIAL4G** |
| **PPIB** |
| **PPIC** |
| **PPP4R4** |
| **PPT1** |
| **PRDX3** |
| **PRDX4** |
| **PRDX6** |
| **PREP** |
| **PRKG1** |
| **PRNP** |
| **PROS1** |
| **PRPH** |
| **PRSS23** |
| **PSAP** |
| **PSAT1** |
| **PSG4** |
| **PSG5** |
| **PSMA1** |
| **PSMA4** |
| **PSMA5** |
| **PSMA7** |
| **PSMB1** |
| **PSMB3** |
| **PSMB6** |
| **PSMB9** |
| **PSME1** |
| **PSME2** |
| **PTGFRN** |
| **PTK7** |
| **PTPRF** |
| **PTPRG** |
| **PTPRK** |
| **PTPRM** |
| **PTX3** |
| **PVR** |
| **PXDN** |
| **PZP** |
| **QPCT** |
| **QSOX1** |
| **RAB10** |
| **RAB12** |
| **RAB14** |
| **RAB15** |
| **RAB1A** |
| **RAB1B** |
| **RAB33B** |
| **RAB39B** |
| **RAB3A** |
| **RAB3B** |
| **RAB3C** |
| **RAB40C** |
| **RAB43** |
| **RAB4A** |
| **RAB4B** |
| **RAB6A** |
| **RAB8B** |
| **RAC1** |
| **RALGAPA2** |
| **RAP1B** |
| **RBP4** |
| **RCN1** |
| **RCN3** |
| **RDX** |
| **RECK** |
| **RFX7** |
| **RGAG1** |
| **RGMB** |
| **RGN** |
| **RNASE4** |
| **RNASET2** |
| **RNF135** |
| **RNH1** |
| **RNPEPL1** |
| **ROBO1** |
| **RSU1** |
| **RTFDC1** |
| **S100A11** |
| **S100A4** |
| **S100A6** |
| **SCN5A** |
| **SDC4** |
| **SEC22B** |
| **SEMA4B** |
| **SEMA5A** |
| **SEMA7A** |
| **SEMG1** |
| **SEMG2** |
| **SENP6** |
| **SEPP1** |
| **SERPINA10** |
| **SERPINA3** |
| **SERPINA7** |
| **SERPINB6** |
| **SERPINC1** |
| **SERPINE2** |
| **SERPINF2** |
| **SERPING1** |
| **SERPINI1** |
| **SEZ6L2** |
| **SFRP2** |
| **SH3BGRL3** |
| **SHBG** |
| **SIAE** |
| **SIL1** |
| **SLC3A2** |
| **SLIT3** |
| **SNAP91** |
| **SOD2** |
| **SPARC** |
| **SPARCL1** |
| **SPEN** |
| **SPP2** |
| **STC1** |
| **STC2** |
| **SULF1** |
| **TAGLN** |
| **TAGLN2** |
| **TALDO1** |
| **TAOK1** |
| **TBL1X** |
| **TFRC** |
| **TGFB1** |
| **TGFB2** |
| **THBS1** |
| **THBS2** |
| **THBS3** |
| **THBS4** |
| **THY1** |
| **TIE1** |
| **TIMP1** |
| **TIMP2** |
| **TIMP4** |
| **TLN1** |
| **TLN2** |
| **TMEM132A** |
| **TNC** |
| **TNFAIP6** |
| **TNXA** |
| **TPM1** |
| **TPM2** |
| **TPM3** |
| **TPM4** |
| **TPP1** |
| **TRAP1** |
| **TSPOAP1** |
| **TUBA1B** |
| **TXN** |
| **TXNDC17** |
| **UBA1** |
| **UBA52** |
| **UBB** |
| **UBE2K** |
| **UBE2N** |
| **UBE3B** |
| **UCHL1** |
| **ULBP2** |
| **VAPA** |
| **VAPB** |
| **VASN** |
| **VAT1** |
| **VCAM1** |
| **VCAN** |
| **VCL** |
| **VCP** |
| **VEGFC** |
| **VIT** |
| **VNN1** |
| **VTN** |
| **WNT5A** |
| **WWC2** |
| **XRCC5** |
| **XXYLT1** |
| **XYLT2** |
| **YWHAB** |
| **YWHAE** |
| **YWHAG** |
| **YWHAH** |
| **YWHAQ** |
| **YWHAZ** |
| **ZNF239** |
| **ZNF486** |

UniProt acronyms of the proteins, identified by mass spectrometry, in the culture medium of human olfactory ecto-mesenchymal stem cells. Using IPA software, molecules associated to cell movement, transmigration and homing were identified. Cells highlighted in green indicate the proteins associated to homing, transmigration and cell movement. In purple are listed the proteins associated to transmigration and cell movement. In blue are reported the proteins associated to homing and cell movement. In orange appear the proteins associated uniquely to cell movement.
