# Supplementary material for: From Blood to Lesioned Brain: An In Vitro Study on Migration Mechanisms of Human Nasal Olfactory Stem Cells
Source: Stem Cells Int. 2017 Jun 18;2017:1478606. doi: 10.1155/2017/1478606 (PMC5494110; doi:10.1155/2017/1478606)
Supplement: Supplementary file 2 [file 1478606.f2.docx]

**Supplementary table 2: Mouse and human target and control probes used in RT-qPCR.**

| **M**  **O**  **U**  **S**  **E** | **Gene** | **TaqMan® Gene Expression Assay probes IDs** |
| --- | --- | --- |
|  | *C3* | Mm00437858_m1 |
|  | *Ccl2* | Mm00441242_m1 |
|  | *Spp1* | Mm00436767_m1 |
|  | *Cxcl10* | Mm00445235_m1 |
|  | *Gfap* | Mm01253033_m1 |
|  | *F4/80 - Emr1* | Mm00802529_m1 |
|  | *Tnf* | Mm99999068_m1 |
|  | *Il1b* | Mm01336189_m1 |
|  | *Gapdh* | Mm99999915_g1 |
| **H**  **U**  **M**  **A**  **N** | *CCR2* | Hs01560352_m1 |
|  | *CCR1* | Hs00928897_s1 |
|  | *CCR4* | Hs00747615_s1 |
|  | *CCR10* | Hs00706455_s1 |
|  | *ABL1* | Hs01104728_m1 |
